# Short Term Advantages of a Public-Private Partnership for Tuberculosis in Guinea Bissau: Reduction of Mortality and Increased Diagnostic Capacity

**DOI:** 10.4084/MJHID.2014.049

**Published:** 2014-07-01

**Authors:** Fina Vieira, Mamadu Saliu Sanha, Fabio Riccardi, Raffaella Colombatti

**Affiliations:** 1 Hospital Raoul Follereau, Bissau, Guinea Bissau, University of Tor Vergata, Rome, Italy; 2 Aid, Health and Development-Onlus, University of Tor Vergata, Rome, Italy; 3 Department of Biomedicine and Prevention, University of Tor Vergata, Rome, Italy; 4 Clinic of Pediatric Hematology-Oncology, Department of Maternal and Child Health, Azienda Ospedaliera-University of Padova, Padova, Italy

## Abstract

**Background:**

Tuberculosis (TB) is widespread in Africa, but weak health systems in developing countries, often display poor quality of care with delays in case identification, irrational therapy and drug shortage, clinical mismanagement, unnecessary expenditures for patients, reduced adherence and increased mortality. Public-private partnership has demonstrated to increase TB case detection, but less is known about its effects on quality of care, mortality and costs for hospitalized TB patients.

**Methods:**

Clinical outcomes and costs for TB patients at the TB National Reference Center of Bissau, in Guinea Bissau, West Africa, were determined during the first 5 months of the public-private management and compared to the ones of previous years when the hospitals was under direct Government’s management.

**Results:**

215 (2009–2010) and 194 (2012–2013) patients were admitted, respectively. Improvement (p<0.05) was observed in mortality reduction (21% vs 6%), cause of death determination (50% vs 85%), treatment abandonment (15 vs 1). Direct costs for patients during TB diagnostic pathway and inpatient care were significantly reduced, 475 vs. 0 USD.

**Conclusions:**

Public-private partnerships displays significant short term benefits in National TB reference centers, even in post-conflict countries. Further studies could aid in determining the overall long term benefits of this type of cooperation, and the specific characteristic of TB and concomitant hematologic and infectious diseases in TB admitted patients.

## Introduction

Tuberculosis (TB) treatment has saved more than 2 million people since 1995.^1^ Nevertheless, too many are not diagnosed, not treated or not properly treated. Public-private partnership has increased Tuberculosis (TB) case detection in different contexts.^2^–^3^

Weak health systems in developing countries, often display poor quality of care, delays in case identification, irrational therapy and drug shortage, unnecessary expenditures for patients and increased mortality.^2^,^3^ Despite a policy of free drug treatment, TB health services in many countries charge all income groups, costs are high and adherence measures are inadequate.^4^–^5^ Patients’ costs can be particularly burdensome for TB affected households in Africa.^6^

Guinea-Bissau, in West Africa, has been experiencing annual military coups since the 1999 civil war. The country’s health indicators are among the worst in Africa.^7^ Proportion of budget spent on health is 2% of GDP, with total dependence on external financial support to the budget of the Health Ministry. The payment of salaries to health workers has often been subject to delay, and morale is poor.^7^

TB incidence is 242/100.000.^1^ Case detection rate for all forms of TB, at 48%, has been declining since it reached 81% in 1995, indicating a decline in programmatic performance.^1^,^7^ The treatment success rate, for smear-positive TB (60%) and other forms (about 55%) is low.^1^

The TB National Program operates through the National Reference Center in Bissau, Hospital Raoul Follereau (HRF), Regional Hospitals in the Regions and TB Health Centers throughout the country. According to the National Guidelines, TB patients in poor clinical conditions or with severe disease are admitted to the HRF, after referral from regional hospitals or health centers.^8^

The National Reference Center for Tuberculosis and Lung Diseases, HRF, is an 115 bed hospital which includes an in-patient service (three words: men, women and children), an outpatient service, laboratory, two X-Ray Units, a pharmacy, service area (kitchen, laundry, ironing) and two cafeterias. Since October 1^st^ 2012 the Health Ministry has entrusted the management of the HRF to the International Organization, Aid, Health and Development (AHEAD). The Government would give TB drugs received from international donors, pay the staff salaries and provide electricity. AHEAD would supply a top-up for the personnel and the diagnostic and treatment pathway for free to inpatients (including exams, non-specific drugs, food). The public hospital would be managed by the private sector, but within the framework of the National TB Program. It was hoped that this public-private partnership would improve the quality of diagnosis and treatment of TB suspects, increase adherence while reducing mortality and costs.

## Methods

According to the National Guidelines (8), as part of routine clinical diagnostic evaluation, patients who are admitted to the HRF with a suspect of TB, received a three sample sputum analysis and a thorax X-Ray. Ziehl-Neelsen’s sputum staining technique is used, and patients are considered smear positive if acid fast bacilli are shown on at least two samples. Sputum Culture and PCR are not available in the country for the present being. Smear negative patients are considered to have TB according to the physician’s evaluation of chest X-Ray and clinical condition. Additional analyses (complete blood count, biochemistry, urine and stool evaluation and culture) are performed if necessary, based on physician’s judgment. TB is treated according to the National Guidelines,^8^ with a four drug regimen for two months (rifampicin, isoniazid, ethambutol and pyrazinamide) followed by two drugs for the following six months (isoniazid and ethambutol); five drugs are used in case of relapsed TB (streptomycin, rifampicin, isoniazid, ethambutol and pyrazinamide).

We evaluated all-cause mortality rates, adherence to the hospitalization phase of TB treatment, diagnostic procedures and direct costs for inpatients in the first 5 months of the public-private management (October 1^st^ 2012–February 28^th^ 2013); furthermore, we compared them to the five months of the same season in previous years (October 1^st^ 2009–February 28^th^ 2010), when complete data were available. Data on TB co-infections or hematologic malignancies and severity score were seldom available and not routinely utilized to grade and classify patients in both periods and therefore could not be compared. No user fees were applied during the public-private management while user fees were applied during previous years under the Government direct management of the hospital both during the diagnostic pathway and for admission. Patients’ clinical cards and hospital registries were used to cross-check diagnosis, therapy and admission-discharge dates. Pearson’s chi-square test was used to compare variables within groups. *p-values*<0.05 were considered statistically significant.

## Results

The majority of patients came from Bissau (82% in 2009 and 71% in 2012); there was a slight increase in patients coming from outside the capital in 2012 (29 % vs 18%).

No treatment abandonment was observed in the two periods, but 15 drop outs were registered between January 2009 and May 2012 –when user fees were applied- and only 1 between June 2012 and May 2013 –when treatment was free.

The clinical characteristics of admitted inpatients are detailed in Table 1.

**Figure 1:**
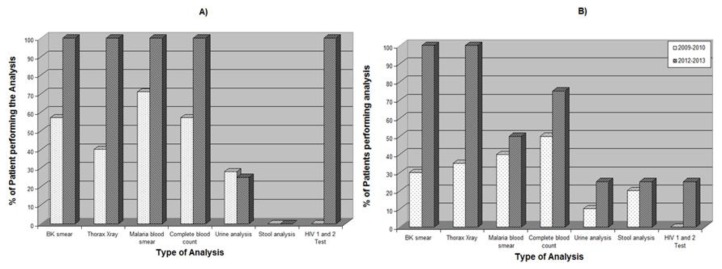
Diagnostic capacity in October 2009–February 2010 vs. October 2012–February 2013 for Hospital Raoul Follereau’s inpatients who died: A) TB patients who died after the first week of admission; B) TB patients who died within one week of admission. p-value was significant (<0.05) for both A) and B)

A significant reduction in mortality (21 vs 6%) was observed in October 2012–February 2013, both during the first week of admission and afterwards (Table 1 and Table 2). Within the population of patients who died, the number of diagnostic exams was significantly different, with improved quality of diagnostic pathway in October 2012–February 2013 compared to October 2009–February 2010 (Figure 1A–B). This allowed a significant increase in the number of patients in which the cause of death could be determined (Table 2).

The number and type of available drugs was also different in the two periods with increased availability of i.v. antibiotics and antimalarials in 2012–2013 compared to 2009–2010 (1.6 vs 0.2 per patient).

Direct costs of TB diagnosis (XRays, BK analysis, Laboratory exams) in October 2009–February 2010 were 31000 CFA per patient (65,11 USD) while in October 2012–February 2013 they were zero. No direct cost for hospitalization was required in October 2012–February 2013 while in October 2009–February 2010 they were estimated in 195000 CFA per patient (409,5 USD) (bed occupation, food, non specific drugs).

## Discussion

Our experience suggests that a public-private partnership within the framework of the National TB Program in a National Reference Center, can be successfully implemented in a low resource and post-conflict country. Even if a six months period is too short to evaluate long term indicators, significant reductions in mortality and improvements in diagnosis can already be observed in the short term.

In-hospital mortality for TB patients is multifactorial and remains high in many countries.^9^ The significant reduction in mortality (21 vs 6%) observed in our cohort was due to the reduction of mortality both during the first week of admission and afterwards. Patients, usually admitted in severe conditions, frequently have disseminated infections or comorbidities. It is likely that patients have sought care earlier in the course of the disease, knowing it was free of charge. Moreover, the increased availability of diagnostic tools, iv antibiotics, antimalarials and saline solutions might have had an impact in the possibility of physicians to manage acutely ill patients and therefore contributed to the mortality reduction both in the first week of admission and afterwards.^10^

No treatment default was observed since the free hospitalization regimen was implemented; meaning that lack of will to be cured is not an issue. TB patients in Africa often default in the hospitalization phase: hospitalization is problematic due to poor general conditions in TB hospitals, costs incurred by patients during hospitalisation and because patients need to earn living or take care of their families.^11^ Stock ruptures, shortage of reagents and drugs and low salaries produce lack of motivation and poor morale impairing health personnel’s work and dramatically reducing diagnostic and treatment performances in Africa. The free care of severely ill TB patients, including drugs and nutritional support, coupled with top-up for the personnel is likely to have contributed to eliminate drop outs and reduce mortality after the first week of admission.

Our short term analysis has several limits. First, we considered all cause mortality and could not detail TB related mortality, mainly due to the limited number of diagnostic exams performed in the first period. It is likely that considering complete blood count and other laboratory exams on a routine basis will allow a better definition of other causes of mortality in TB inpatients such as acute anemia, concomitant infections, hematologic malignancies, etc. Second, the direct cost that were evaluated included the costs that are necessary for TB diagnosis and inpatient care and not all the direct cost that the patients experience, therefore they are probably underestimated and overall direct costs are likely to be higher in both periods. Third, a short term evaluation of public private partnership in only five months does not allow detailing all the benefits and drawbacks of such a collaboration. Nevertheless, post conflict and low resource countries such as Guinea-Bissau that do not seem able to find rational and appropriate ways to come out of isolation and to tackle health challenges, desperately need positive experiences. We think it’s worth reporting the improvement obtained in a short period of time of a public-private international partnership in order to move forward and enhance TB diagnosis, definition of co-morbidities, treatment and care in the long term.

## Conclusion

The main challenge in fighting the TB epidemics in Sub-Saharian Africa is to improve the sanitary system. Our experience demonstrates that international public-private partnerships in TB hospital settings can contribute, in the short term, to increase adherence to the hospitalization phase of intensive treatment, improve quality of diagnosis and care and reduce mortality through a free pathway of diagnosis and care.^12^

## Figures and Tables

**Table 1:** Main clinical characteristics and outcomes of admitted inpatients to HRF

	October 2009–February 2010		October 2012–February 2013		p-value

	N	%	N	%	

**Admissions**	215	100	194	100	

**Gender**					
M	132	61	110	57	0.6
F	83	39	84	43	

**Mean Age** (years)	39		32		0.9

**Diagnosis of TB at admission** (%)					
New Diagnosis	175	81	167	86	0.2
Relapse	5	2	6	3	
Not Known	35	17	21	11	

**Type of TB at admission**					
Pulmonary TB Smear +	100	47	122	63	0.1
Pulmonary TB Smear −	54	25	5	2	
ExtraPulmonary TB	11	5	16	8	
Other	50	23	52	27	

**HIV status**					
*Positive*	*66*	*31*	*82*	*42*	0.05
HIV1	40	19	49	25	
HIV2	6	3	5	3	
HIV1+2	20	9	28	14	
*Negative*	*92*	*43*	*104*	*54*	
Not done	57	26	8	4	
On treatment	0	0	7	4	

**Deaths**	46	21	12	6	0.001
In the first week	24/46	53	4/12	33	
After the first week	22/46	47	8/12	67	

**Discharges**	149		154		0.2

**Death/Discharge Ratio**	0.34		0.09		0.03

**Table 2:** Main clinical characteristics of inpatients who died, during the first week of admission and after the first week of admission

	Patients who died during the 1^st^ week		Patients who died after the 1^st^ week		p-value

	October 2009–February 2010	October 2012–February 2013	October 2009–February 2010	October 2012–February 2013	

**Number of Deaths**	24	4	22	8	0.01

**M:F**					

**Mean Age** (years)	39.3	31.2	44	31	0.1

**Mean Lenght of Stay** (days)	3	0.75	30.4	20.5	0.04

**TB Diagnosis at admission**					
Pulmonary TB Smear +	4	3	4	4	
Pulmonary TB Smear −	3	1	3	4	0.01
ExtraPulmonary TB	-	-	-	-	
Other	4	-	-	-	
Not known	12	-	15	-	

**Symptoms at admission**					
Fever	6	2	5	4	0.001
Convulsions	1	-	-	-	
Respiratory distress	4	2	1	2	
Other	-	-	2	2	
Not Known	13	-	37	-	

**Clinical Diagnosis of death**					
Hemoptysis	-	1	-	1	
Cardio-respiratory failure	2	2	3	1	0.002
Malaria	2	-	1	1	
Fever	4	1	4	2	
Cancer (Kaposi Sarcoma)	-	-	2	-	
AIDS	-	-	-	3	
Not Known	18	-	34	-	
